# Early integration or last consultation: in-house palliative care involvement for hospitalized patients in tertiary medicine—a retrospective analysis

**DOI:** 10.1007/s00520-025-09312-0

**Published:** 2025-03-05

**Authors:** Nico Bucklar, Markus Schettle, M. Feuz, F. Däster, Sebastian M. Christ, David Blum, Caroline Hertler

**Affiliations:** 1 University of Zurich, Zurich, Switzerland; 2 Competence Center Palliative Care, Department of Radiation Oncology, University Hospital Zurich, Rämistrasse 100, 8091 Zurich, Switzerland; 3 Department of Radiation Oncology, University Hospital Zurich, Zurich, Switzerland

**Keywords:** Specialist palliative care, Consultation, Early integration, Interprofessional, Patient needs

## Abstract

**Background:**

The importance of timely integration of palliative care has been confirmed over the past years for any patient suffering from a life-threatening or life-limiting disease. Palliative and supportive care increases quality of life of patients and caregivers in both oncological and non-cancer diseases and should therefore be offered on a needs-based approach and throughout the disease trajectory.

**Methods:**

We analyzEd all in-patient consultation requests of the leading university hospital in Switzerland in 2019. Sociodemographics, symptoms, and specific requests as well as provided support offers were retrieved from the electronic patient files. Demographic and clinical data was analyzed by descriptive statistics between groups. Overall survival from diagnosis and time from consultation to death was analyzed by means of Kaplan–Meier estimates and log-rank test.

**Results:**

We identified 507 in-patient consultation requests from 24 oncological and non-oncological departments in 2019. The final analysis cohort comprised 290 patients, of which 133 women (45.9%). Median overall survival of the population from diagnosis was 21.1 months (CI 15.57–26.72). Median survival from palliative care consultation was 29 days (CI 20.89–37.11), independent of primary diagnosis (*p* = 0.298) or sex (*p* = 0.079). A total of 38.9% (*N* = 140) of consultations were requested concurrently to a tumor-targeted treatment. Palliative care consultations provided more support services than requested (*p* < 0.001).

**Conclusion:**

Our findings underline the persisting late involvement of palliative care services in the disease trajectory, despite being a concurrently consultable and readily available support service to address patient and caregiver needs.

## Introduction

The goal of palliative care is the holistic care for persons with a life-limiting or life-threatening disease, including the improvement of the quality of life of patients and their loved ones and the relief of suffering in all dimensions (WHO). While the origins of palliative care arose from hospice and end-of-life care, the value of early integration of palliative care gained importance over the past decade. Symptom load can be challenging and stressful for patients even in early stages of disease, which is why oncological societies as the American Society for Clinical Oncology (ASCO) reinforced the importance of early concurrent palliative care in oncology in their guidelines [1–3]. Meanwhile, non-cancer associations have developed guidelines for integration of palliative care in their fields [4–6], underlining the value of supportive and concurrent care in non-oncological disease as well.

The benefit of palliative interventions beyond hospice care was confirmed in early disease management and through several clinical studies, improving quality of life of patients and caregivers [7, 8]. At the same time, futile and potentially harmful treatments are reduced. However, physicians and patients are still reluctant to introduce specialist palliative care early in the course of disease out of fear it might be a bad predictor or shorten survival. This has been disproven by a large, randomized study demonstrating that early introduction of palliative care not only improved quality of life and lowers depressive symptoms, but also prolonged overall survival in patients with lung cancer [9, 10]. In this context, the recent terminology shift from *early* to *timely* integration of palliative care in a stepped approach—reflecting patients’ needs at any time during the course of disease, and possibly in several steps, more than the disease trajectory itself—may be helpful to break barriers that form obstacles to a beneficial and targeted supportive palliative care approach [11]. However, a recent meta-analysis on the integration of specialist palliative care services either as in-patient or out-patient services found that, while specialist palliative care influenced quality of life, especially in cancer patients at early stages of the disease, none of the interventions were triggered by patients’ needs [12].

Despite the increasing evidence of beneficial palliative care integration as concurrent care and multiple guideline recommendations from important associations and societies [2, 3, 13–15], including an international consensus paper on referral criteria for outpatient specialist palliative cancer care [16], a timely integration is still not an established approach, and referral to palliative care usually takes place late in the course of disease, despite substantial symptom burden of patients and families. Several promising approaches for optimizing timely and early integration have been developed lately, including Goals of Care Programs in cancer centers or Trigger Tools to bridge oncology outpatient services with palliative care referral [17, 18]. We previously published the experience of an attempt to early integration of palliative care through an outpatient palliative care service [19]. In the setting of ambulatory patients—presumably early in their course of disease—referral to a first specialist palliative care contact occurred in median 3 months before death, irrespective of the primary disease (cancer vs. non-cancer) and of the length of disease trajectory. This is not congruent to the recommendations that palliative care should be offered within 8 weeks from diagnosis of advanced disease. Likewise, Adamidis et al. recently demonstrated a median time from any palliative care consultation to death of 17.2 days at the largest tertiary hospital in Austria [20]. Late referral however translates into little to no time to implement support systems and provides appropriate palliative care offers.

In this light, we analyzed the in-house consultations at the University Hospital Zurich, Switzerland, a large tertiary hospital, to define time to referral and to identify factors involved in specialist palliative care consultation requests.

## Methods

### Data source

We conducted a retrospective cohort study including patients hospitalized on any other ward than the specialized palliative care unit at the University Hospital of Zurich (USZ) in 2019, and for whom an in-house palliative care consultation was requested. The primary goal was to characterize the main reasons for referral and to define the referral time point in the disease trajectory. A total of 507 in-patient consultations were requested over a 12-month period. The data analysis excluded 147 consultations from patients that did not provide consent for data analysis. All included patients consented to coded use of data. Patient data was retrieved from the electronic medical record system. Data on patient demographics, primary hospitalization department, primary disease and clinical characteristics were collected. We also collected information on advance directive, patient network, and relatives. Pain was scored by numerical rating scale (NRS) from 0 (no pain) to 10 (maximal pain); self-care capacity was classified by the nurse-rated functionality score “Selbstpflegeindex” (SPI) rated from 10 (fully incapacitated) to 40 (fully capable to self-care). Finally, we documented the date of initial diagnosis, the date of in-house consultation, the dates of death, and the date of last contact, respectively.

### Characteristics of the in-patient consultation service

The palliative in-patient consultation service at the USZ was established in 2012 to ensure that any hospitalized patient at the University Hospital may have access to palliative care, even when not hospitalized on the specialized palliative care unit (SPCU). As part of the University Hospital Zurich with 900 beds and approximately 42,000 in-patients per year, the in-patient consultation service provides consultation and information on any palliative care–related topic for many patients and their caregivers that could or should not be hospitalized primarily on the SPCU. The in-patient consultation service is an interprofessional team of a specialized palliative care nurse and a palliative care physician that covers all departments upon request.

### Statistics

Demographic and clinical data was analyzed by descriptive statistics. We calculated mean and standard deviation for all continuous variables. The chi-square test was performed for analysis of nominal variables, and the Mann–Whitney *U* test was used for the comparison of ordinal variables between groups. Survival was calculated from time of consultation to death, overall survival was calculated from time of diagnosis to death, and survival was analyzed by Kaplan–Meier estimates. Kaplan–Meier curves were compared using the log-rank test. Patients without event (death) were censored at time of last follow-up. Statistical significance was set at *p* < 0.05. For statistical analysis, SPSS Version 28 was used (SPSS IBM Corp., Armonk, NY, USA).

### Ethics

This study as part of a larger project on palliative care patients was approved by the Swiss Cantonal Ethics Committee before initiation of the project (BASEC ID #2019–02488) and was conducted in accordance with the Declaration of Helsinki and applicable regulatory requirements. Consent to use of coded data was based on written documented general consent in the internal electronic health record of the University Hospital Zurich, allowing for anonymous retrospective data analysis within a research project approved by the competent authorities.

## Results

### Patient- and sex-related aspects

**Fig. 1:**
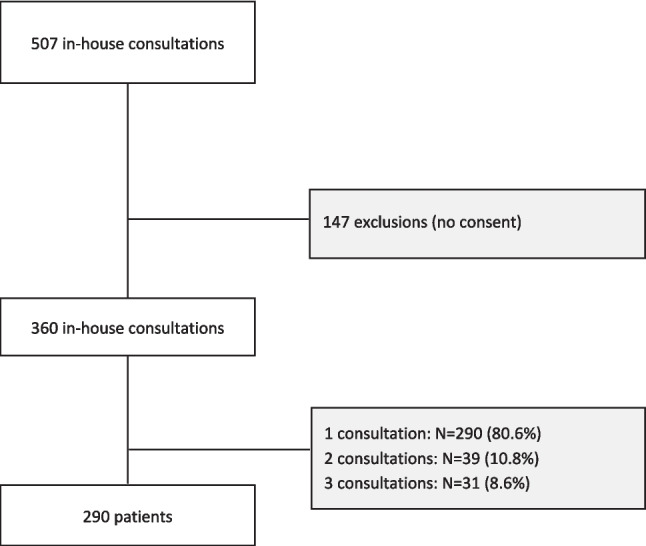
In-house consultation and patient selection

We identified 507 in-patient consultation requests, of which 147 were excluded from the analysis due to the lack of given consent. In the final sample size, 290 patients, of which 133 women (45.9%), were seen in 360 consultations (Fig. 1). All consultations were the first contact with specialist palliative care for the patients. Mean patient age was 65.2 (range 19–101). Most of the patients were Swiss (*N* = 225; 77.6%); were married (*N* = 166; 57.2%), with a general insurance (*N* = 233; 81.9%); and suffered from a cancer diagnosis (*N* = 241; 83.1%). There was no disparity regarding age or main disease cluster in this cohort. Men reported their partner as primary contact more often than women, who were also often represented by children, friends, or siblings (*p* = 0.050). Further patient characteristics are summarized in Table 1.

**Table 1:** Patient group comparison by sex

	Male (*N* = 157) *N* (%)	Female (*N* = 133) *N* (%)	*p*-value
Age (years) Median Range	68.0 27–95	65.0 19–101	0.683
Age groups < 50 50–69 70–79 > 80	16 (10.2) 65 (41.4) 43 (27.4) 33 (21.0)	24 (18.0) 54 (40.6) 32 (24.1) 23 (17.3)	0.225
Disease Oncological Non-oncological	132 (84.1) 25 (15.9)	109 (82.0) 24 (18.0)	0.372
Length of stay (days) ≤ 1 2–7 8–14 15–30 > 30 days	0 (0) 33 (21.0) 49 (31.2) 44 (28.0) 31 (19.7)	3 (2.3) 20 (15.0) 44 (33.1) 51 (38.3) 15 (11.3)	**0.031**
Next relative None Partner Child Friend Sibling Parent Other	2 (1.3) 103 (65.6) 28 (17.8) 3 (1.9) 12 (7.6) 8 (5.1) 1 (0.6)	0 (0) 68 (51.1) 37 (27.8) 8 (6.0) 14 (10.5) 4 (3.0) 2 (1.5)	**0.050**
Discharge to Home Institution Hospice Palliative hospital (external) Other hospital Rehabilitation Death on ward	42 (26.8) 24 (15.3) 4 (2.5) 7 (4.5) 3 (1.9) 3 (1.9) 74 (47.1)	51 (38.3) 16 (12.0) 7 (5.3) 6 (4.5) 6 (4.5) 5 (3.8) 42 (31.6)	0.067
Pain levels (NRS) 0–3 4–6 7–10 Missing	59 (59.6) 30 (30.3) 10 (10.1) 58	59 (63.4) 23 (24.7) 11 (11.8) 40	0.675

Significant *p*-values are indicated in bold

### Disease-related aspects

**Fig. 2:**
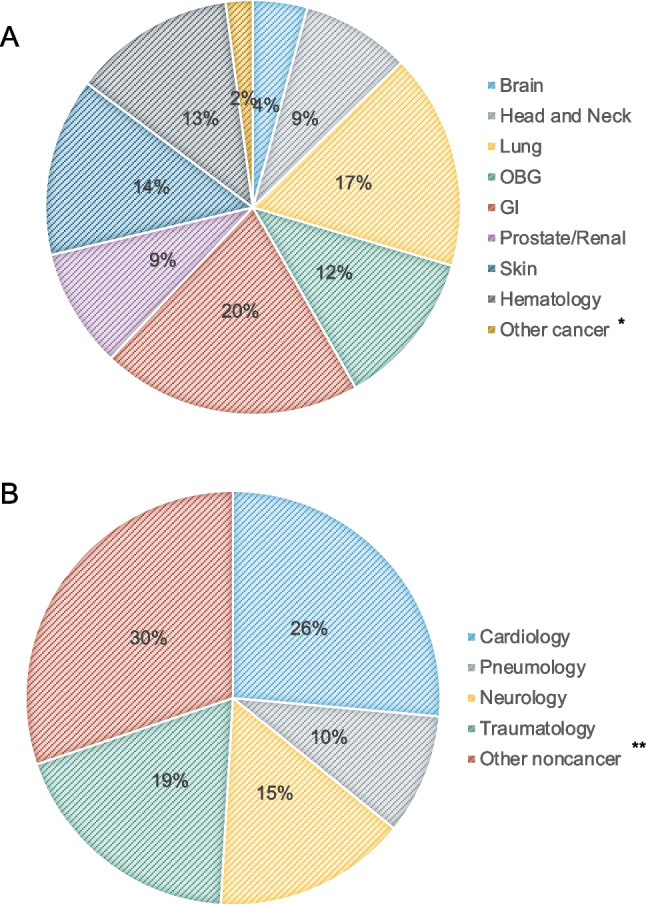
Main primary diagnosis in **A** cancer and **B** non-cancer patients. * comprises cancer of unknown origin. ** comprises infectious diseases, psychiatric diseases, rheumatologic diseases, geriatric diseases, and vascular diseases

Requests came from 24 different departments in-house and included oncological as well as non-oncological services. Most patients suffered from an oncological disease (*N* = 241; 83.1%), the most common tumor entities being gastro-intestinal tumors (20%), lung (17%), skin cancer (14%), and hematological cancer (13%). Non-cancer diagnoses comprised diseases from cardiology (26%), traumatology (19%) neurology (15%), and pneumology (10%). Other non-cancer diseases comprised infectious diseases, vascular diseases, and psychiatric diseases, among others (Fig. 2). A total of 38.9% (*N* = 140) of consultations took place concurrently with a tumor-targeted treatment, of which 55% (*N* = 77) chemotherapy, 30% (*N* = 42) radiation treatment, 28.6% (*N* = 40) immunotherapy, and 9.3% (*N* = 13) surgery.

Cancer patients in this cohort were usually younger (median age 64.0 vs. 73.0), including significantly more persons in the group of patients < 50 years (*p* = 0.046). There were no significant differences between the groups regarding length of stay (*p* = 0.725), discharge location (*p* = 0.097), or next relative (*p* = 0.209). However, when compared to cancer patients, non-cancer patients had a significantly worse ECOG performance status 3–4 (88.0% vs. 53.1%; *p* < 0.001) and a worse SPI < 20 (55.1% vs. 15.8%; *p* < 0.001) compared to cancer patients.

### Consultation requests

The majority of patients was seen one time during hospitalization (*N* = 290, 80.6%), 39 patients (10.8%) had 2 consults, and 31 patients (8.6%) were seen 3 times. Consultations were requested in median 9.76 days after admission to the hospital (range 0–138 days), with a median length of stay of 15.0 days (range 0–181 days).

The main reasons for in-patient palliative care consults were (1) requests to optimize physical symptoms (34.7%, *N* = 125), (2) requests for transition to the specialized palliative care unit (SPCU) (45%, *N* = 162), (3) requests for social support/organization of post-hospitalization support services (37.2%, *N* = 125), (4) end-of-life questions including assisted suicide wishes or requests for palliative sedation (0.6%, *N* = 2), and (5) support with advanced directives (6.4%, *N* = 23). The median number of requests per consult was 2 (range 1–4). The median time of consultation was 50 min (range 5 min–85 min).

Regarding completion of assignment, the palliative care consultation usually provided more consultations on each topic than requested. Physical symptoms and social support services were discussed more often than anticipated with the patients (*p* < 0.001); end-of-life topics (*p* = 0.017) and advance directives (*p* = 0.006), which were rarely requested, were also addressed more than requested within the palliative care consultation (Table 2). Consultation requests for men comprised the wish for social support post-hospitalization (*p* = 0.012) significantly more often than for women. Interestingly, the palliative care consultation service provided information on support services to men (69.4%) and women (66.7%) equally, despite the lack of request (*p* = 0.329).

**Table 2:** Consultation group comparison by sex—requested vs. provided consultation topics

Consult reasons	Requested			Provided			Requested vs. provided
	Male (*N* = 183) *N* (%)	Female (*N* = 177) *N* (%)		Male (*N* = 183) *N* (%)	Female (*N* = 177) *N* (%)		
Physical symptoms Yes No	68 (37.2) 115 (62.8)	57 (32.2) 120 (67.8)	0.190	127 (69.4) 56 (30.6)	115 (65.0) 62 (35.0)	0.050	** < 0.001**
Transfer to SPCU Yes No	90 (49.2) 93 (50.8)	72 (40.7) 105 (59.3)	0.065	91 (49.7) 92 (50.3)	66 (37.3) 111 (62.7)	**0.011**	** < 0.001**
Social requests/post-hospital situation Yes No	79 (43.2) 104 (56.8)	55 (31.1) 122 (68.9)	**0.012**	127 (69.4) 56 (30.6)	118 (66.7) 59 (33.3)	0.329	** < 0.001**
End-of-life topics Yes No	1 (0.5) 182 (99.5)	1 (0.6) 176 (99.4)	0.742	33 (18.0) 150 (82.0)	15 (8.5) 162 (91.5)	**0.006**	**0.017**
Advance directives Yes No	10 (5.5) 173 (94.5)	13 (7.3) 164 (92.7)	0.304	37 (20.2) 146 (79.8)	34 (19.2) 143 (80.2)	0.366	**0.006**

Significant *p*-values are indicated in bold

Transition to the specialized palliative care unit (SPCU) was requested in 162 consultations (45.0%). Patients for which a transition to SPCU was requested had a poorer ECOG performance status 3–4 (*p* = 0.020) and a lower SPI < 20 (*p* < 0.001) compared to those for which a transition was not requested. Pain levels were reportedly higher in those patients, yet without reaching significance (15.8% vs. 7.8%, *p* = 0.218). A total of 157 transitions (43.6%) eventually took place.

### Survival

**Fig. 3:**
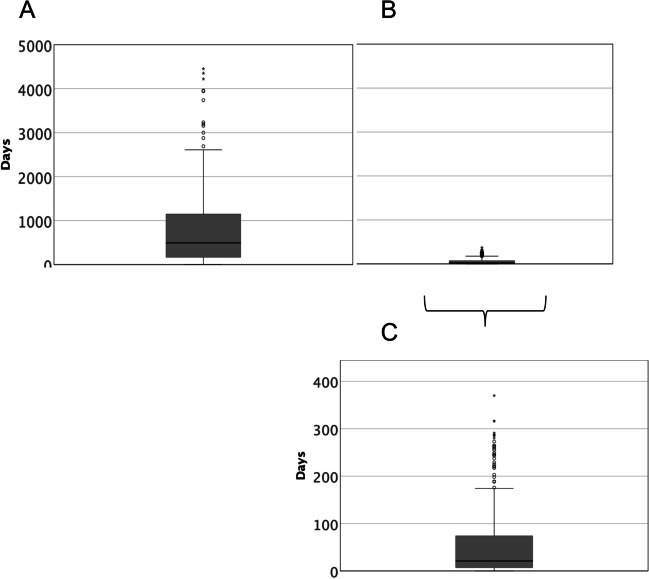
Survival whole cohort **A** from diagnosis to death; **B** from consultation to death; and **C** zoomed-in scale of survival from consultation to death

Median overall survival of the population was 21.1 months (CI 15.57–26.72), with women surviving slightly longer than men (28.19 months vs 19.64 months, *p* = 0.078). Survival from palliative care consultation was 29 days in median (CI 20.89–37.11) independent of the primary disease (*p* = 0.298) or sex (*p* = 0.079) (Fig. 3). Oncological patients were seen in median 27 days before death (CI 18.26–35.74) and non-cancer disease patients 29 days before death (CI 2.79–55.21) (*p* = 0.298). Patients dying in hospital care died in median within 6 days (CI 4.14–7.86) after first palliative care consultation, whereas patients discharged to either home care, nursing home care, or rehabilitation died in median within 73 days (CI 51.65–94.35) after first palliative care consultation (*p* < 0.001). Patients transferred to the specialist palliative care unit after consultation died in median within 13 days (CI 9.30–16.70), compared to those remaining on the general ward (43 days (CI 29.79–56.21)) (*p* < 0.001).

## Discussion

Early integration of palliative care has proven to benefit patients and their families, to reduce unwanted and potentially harmful treatment, and to reduce medical costs [12, 21, 22]. However, the appropriate time to integrate is not well defined yet. After the shift from being mainly associated with hospice care to attempting early integration of palliative care, the latest approach comprises a timely and stepped integration of palliative care based on patients’ needs more than disease trajectory timelines [11]. Indisputably, a needs-based integration of specialist palliative care, possibly in addition to equally important general palliative care interventions, starting early in the course of disease, will allow for appropriate support system set-ups and for the patients to benefit from them [23]. Several associations and societies recommend a first contact within 2 months from diagnosis of an advanced disease, as well as a first discussion about advanced care planning if survival is supposed to be less than 1 year [2, 3].

We previously published an ambulatory referral time to outpatient specialist palliative care with a median of 3 months before death, already considered late in light of current recommendation for timely integration in cancer and non-cancer patients [19]. Here, we assessed palliative care consultations of patients hospitalized in other departments of the University Hospital and report a referral time of 29 days before death (CI 20.89–37.11) (Fig. 3). On the one hand, this reflects the continuing synonymization of palliative care and end-of-life care, leading to specialist palliative care team involvement for complex late-stage end-of-life care and patient transfer in hospital settings. Accordingly, we found the cohort of patients transferred to the specialist palliative care unit dying significantly earlier than those remaining or being discharged from general wards (13 days vs. 43 days, *p* < 0.001). On the other hand, these results underline the urgent need to define triggers for early referral in already hospitalized patients and to implement those trigger tools in daily clinical work. Disease- or setting-specific trigger screening tools and scores for physicians and nurses, but also artificial intelligence-based tools, have proven to identify needs that warrant palliative care referral from intensive care units, emergency rooms, cancer wards, or non-cancer departments [24–28]. Additionally, trigger assessment should ideally take place before any emergency admission or unplanned hospitalization, considered one of several indicators of aggressiveness of care at the end-of-life in oncology that contradicts a good end-of-life care [29, 30]. Another approach to improve earlier in-patient referral could be the implementation of out-patient interdisciplinary integrated specialist palliative care consultations, which simplifies low-threshold access to the specialist palliative care team during any subsequent hospitalization [31]. We demonstrated earlier the implementation of palliative care expert representatives in six selected oncological and non-oncological departments as a low-threshold liaison to specialist palliative care [32].

Here, requests came from 24 different departments, demonstrating a broad range of patients and disease trajectories. While 83.1% of patients suffered from oncological diseases, consultation requests also came from diverse non-oncological departments as well, including neurology, cardiology, but also psychiatry and infectious diseases, indicating the broad spectrum of needs that needed to be catered to (Fig. 2). Non-cancer patients were older (*p* = 0.046) and had a worse performance status (*p* < 0.001) and a worse ability to self-care (*p* < 0.001) compared to cancer patients, while no difference in overall survival (*p* = 0.876) was observed between both disease groups. The differences in clinical characteristics between cancer and non-cancer patients point toward the fact that those two groups are distinct and might request tailored palliative care interventions. Therefore, while palliative and supportive care benefits both cancer and non-cancer patients, a stepped approach to integration might look differently in those groups.

The common denominator in the two groups however was the timing of palliative care integration. Regardless of the main diagnosis, patients were referred to a palliative care in-house consultation in median 29 days before death (CI 20.89–37.11) (Fig. 3). In light of the recommendations from ASCO or ESMO, stating that all cancer patients should be offered palliative care within 8 weeks from diagnosis of advanced disease, including discussion of advance care planning with patients with a life expectancy below a year, our patient cohort was referred obviously at a very late stage of their disease [2, 3, 13]. Our results are in line with the recently published results from a similar analysis in the largest University Hospital in Austria, identifying a median referral to palliative care of 17.2 days before death [20]. Late referral, however, diminishes the options of any palliative care team to implement solid support systems for either patients or families [20]. While palliative and supportive care may help to stabilize patients and families emotionally by reducing anxiety and distress and unwanted treatment in the end of life, those outcomes may not be fully achieved if provided only in the last month of life [33–35]. However, the fact that approximately 1/3 of patients were seen by the specialist palliative care team while under tumor-targeted treatment should be considered encouraging regarding early integration in cancer patients.

With regard to sex disparity, we did not identify major differences in outcomes. While women did survived longer than men (28.19 months vs 19.64 months, *p* = 0.078), the survival difference did not reach significance. Likewise, time from palliative care consultation to death did not differ significantly between women and men (35 days vs. 19 days, *p* = 0.079), even though women seemed to be referred slightly earlier.

We could confirm that women were more often represented or cared for by children, siblings, or friends compared to men, who were significantly more often cared for by their partner (Table 1). This is in line with data indicating that women in palliative care settings are less likely to be married and more likely to be alone in the end-of-life phase [36]. However, we did not see a difference in reported pain levels (*p* = 0.675), in contrast to previous publications describing higher pain levels and undertreatment for pain in women [37, 38] (Table 1). We found that the palliative care consultation requests comprised the request for social support significantly more often for men than for women (*p* = 0.012), which is in line with current literature indicating that women have less expectation to receive home care, are more often transitioned to nursing homes, and are less often offered support compared to men [36, 39, 40]. Interestingly, despite those differences in requests, the palliative care consultation team eventually provided social support to men and women equally (*p* = 0.329), annihilating the sex differences. Eventually, the consultations provided additional support and information to all patients, compared to the number of requests, underlining the comprehensive and multidimensional duty of palliative care interventions and confirming the added value of concurrent palliative care consultations throughout the broad in-house palliative patient population.

Limitations of the study include the retrospective analysis of the cohort and the lack of patient-reported and caregiver-reported outcomes, which would have been relevant in the context of patient-centered evaluation. Also, a generalization of the results might be precluded by the fact that our patient population comes from a university hospital, therefore prone to comprise far advanced, complex, or otherwise selected patients, as clinical study participants. However, we were able to include a broad spectrum of patients with different oncological, but also non-cancer diagnoses, from one of the largest University Hospitals in Switzerland, and could confirm that late referral remains a fact independent of the primary diagnosis or sex, despite all efforts of timely and early integration.

## Conclusion

Because they are referred in the last phase of life, patients and their families will keep associating palliative care with the last phase of life. Likewise, physicians may remain reluctant to request consultations if they do not see a positive outcome, outcome that may not be provided in time before death due to late referral. To break this barrier, we must develop and increase collaborations with the primary physicians, screen for patient needs at any time of the disease trajectory, educate and inform society about the primary goals and aims of palliative care interventions, and ally as palliative care community to collect outcome data to underline the potential of our efforts.

## Competing Interests

The authors declare no competing interests.

## Data Availability

No datasets were generated or analysed during the current study.
